# The Quiescent Cellular State is Arf/p53-Dependent and Associated with H2AX Downregulation and Genome Stability

**DOI:** 10.3390/ijms13056492

**Published:** 2012-05-24

**Authors:** Ken-ichi Yoshioka, Yuko Atsumi, Hirokazu Fukuda, Mitsuko Masutani, Hirobumi Teraoka

**Affiliations:** 1 Division of Genome Stability Research, National Cancer Center Research Institute, 5-1-1 Tsukiji, Chuo-ku, Tokyo 104-0045, Japan; E-Mails: yuatsumi@ncc.go.jp (Y.A.); mmasutan@ncc.go.jp (M.M.); 2 Department of Biosciences, School of Science, Kitasato University, 1-15-1 Kitasato, Minami-ku, Sagamihara 228-8555, Japan; 3 Division of Cancer Development System, National Cancer Center Research Institute, 5-1-1 Tsukiji, Chuo-ku, Tokyo 104-0045, Japan; E-Mail: hfukuda@ncc.go.jp; 4 Department of Pathological Biochemistry, Medical Research Institute, Tokyo Medical and Dental University, 2-3-10 Kandasurugadai, Chiyoda-ku, Tokyo 101-0062, Japan; E-Mail: tera-pbc@tmd.ac.jp

**Keywords:** cancer, immortality, senescence, genome stability, tetraploidy, H2AX, Arf/p53

## Abstract

Cancer is a disease associated with genomic instability and mutations. Excluding some tumors with specific chromosomal translocations, most cancers that develop at an advanced age are characterized by either chromosomal or microsatellite instability. However, it is still unclear how genomic instability and mutations are generated during the process of cellular transformation and how the development of genomic instability contributes to cellular transformation. Recent studies of cellular regulation and tetraploidy development have provided insights into the factors triggering cellular transformation and the regulatory mechanisms that protect chromosomes from genomic instability.

## 1. Introduction

**Figure 1:**
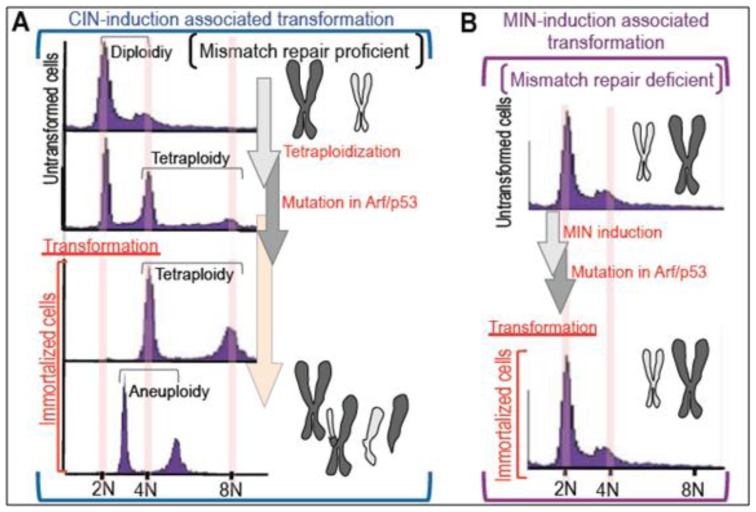
Development of genomic instability prior to cellular transformation. Most cancers that arise in aging organs and cells transformed *in vitro* develop due to mutations, such as those in the Arf/p53 module, and genomic instability, with either chromosomal instability (CIN) (**A**) or microsatellite instability (MIN) (**B**). Whereas MIN is associated with a mismatch repair-deficient background, CIN can also develop on a normal genetic background. In normal mouse embryonic fibroblast cells (MEFs), tetraploidy develops prior to the acquisition of immortality and causes mutations in the Arf/p53 module. Immortalization of MEFs is initially associated with tetraploidy and eventually changes to aneuploidy. By contrast, mismatch repair-deficient cells maintain chromosomal stability during transformation, but MIN develops in the transformed cells.

During cancer development, cells acquire immortality in association with the development of genomic instability [1–3] and mutations in certain genes including those of the Arf/p53 pathway [4–7]. Except for certain tumors associated with specific chromosomal translocations [8], such as infant leukemia and sarcoma [9–13], most cancers that develop at an advanced age [14–22] are characterized by an unstable genome, with either chromosomal instability (CIN) or microsatellite instability (MIN) [23], and specific mutations [24] (Figure 1). Although MIN usually develops on a mismatch repair (MMR)-deficient background [25–29], CIN frequently develops in the presence of a functional MMR system [23]. While cancer cells with MIN are rarely associated with aberrant chromosomes, cancer cells with CIN are characterized by a diversity of chromosomal abnormalities such as aneuploidy; chromosome-loss, -translocation, and -gain; gene amplification; and loss of heterozygosity [30,31]. Similar to the process of *in vivo* carcinogenesis, cells immortalized *in vitro* show genomic instability with either CIN or MIN and mutations in the Arf/p53 module [32]. Furthermore, CIN is inducible on a normal genetic background [33]. These *in vitro* findings illustrate the critical role of genomic instability and loss of Arf/p53 function in the acquisition of immortality, and raise the following critical questions: how is genomic instability induced and how does it contribute to cellular transformation? What is the role of the Arf/p53 module in cancer suppression? This review examines recent evidence regarding the oncogenic stress-induced development of tetraploidy and the role of the Arf/p53 module in suppressing cellular transformation.

## 2. Massive Genomic Rearrangements during Cellular Transformation are Associated with Tetraploidization

Recent advances in DNA sequencing have helped to identify genomic rearrangements associated with tumorigenesis and have revealed the diversity of cancer cell genomes [30,34–37]. Although genomic rearrangements and mutations in cancer have traditionally been thought to accumulate gradually over time, a recent report analyzed cancer cells with complex rearrangements and showed that massive genomic rearrangements can occur during a single catastrophic event [37–40]. Although the exact mechanisms underlying such catastrophic events are still unclear, the accumulated findings suggest that one such event could be associated with tetraploidization. In fact, tetraploid cells have been documented in the early stages of colorectal, breast, and cervical cancer [41,42] and also precancerous lesions [43]. By contrast, the genomes of most malignant cancer cells with CIN are characterized by aneuploidy. Therefore, after massive genomic rearrangement in association with tetraploidization, transformed cells might continuously change the chromosomal status to become aneuploid [44].

Oxidative stress-induced senescence in normal mouse embryonic fibroblast cells (MEFs) [45] can lead to the acquisition of immortality [46] and mutations in the Arf/p53 module [32] as well as CIN [33], in a process analogous to that observed during cancer development. In agreement with the hypothesis that tetraploidization is one of the catastrophic events that triggers massive genomic rearrangements, MEF immortality occurs with the acquisition of tetraploidy and mutation of the Arf/p53 module [33] (Figure 1A). In addition, immortalized tetraploid MEFs eventually become aneuploid during serial cultivation [33,47], similar to the changes observed in cancer cells [44]. Identical tetraploidization and the subsequent aneuploidization were also observed in some other models [48,49]. Thus, “tetraploidy development” is likely involved in the events triggering cellular transformation and the ensuing genomic instability. Importantly, as tetraploidy is observed in the *in vivo* precancerous states [50], *in vitro* tetraploidization occurs prior to the acquisition of immortality, during a period in which MEFs rarely proliferate and inevitably exhibit accumulated γH2AX foci and a senescent appearance, *i.e.*, a flattened and enlarged morphology [33].

Although multiple mechanisms of tetraploidy development have been reported [44], the main tetraploidization process leading to cancerous transformation is most likely a failure of chromosome-bridge-mediated cytokinesis [51–53], which primarily results in bi-nucleated tetraploidy [33,54]. This is because (1) chromosome-bridge formation is associated with DNA lesions induced by oncogene acceleration and aberrant growth activation during pre-cancerous stages [55,56]; and (2) other tetraploidization processes, such as cell-to-cell fusion and mitotic slippage-mediated tetraploidization [57–60] do not induce mutations and massive genomic rearrangements in a single catastrophic event (chromothripsis) [37–40]; however, chromosome-bridge mediated tetraploidization does. In fact, the process of chromosome-bridge mediated tetraploidization is associated with DNA damage under a repair defective background, directly inducing aberrations in the genome. Oncogenic stress can be reproduced *in vitro* by oncogene activation and exogenous growth stimulation due to accelerated S phase entry and the resulting DNA replication stress [55,56]. Importantly, cells subjected to oncogenic stress develop tetraploidy [33] despite being under the opposing influences of cancer progression, reflected by senescence and apoptosis induction [61]. In agreement with the argument supporting aging-associated cancer development with CIN, many senescent cells and aging organs show persistent DNA damage [62,63].

**Figure 2:**
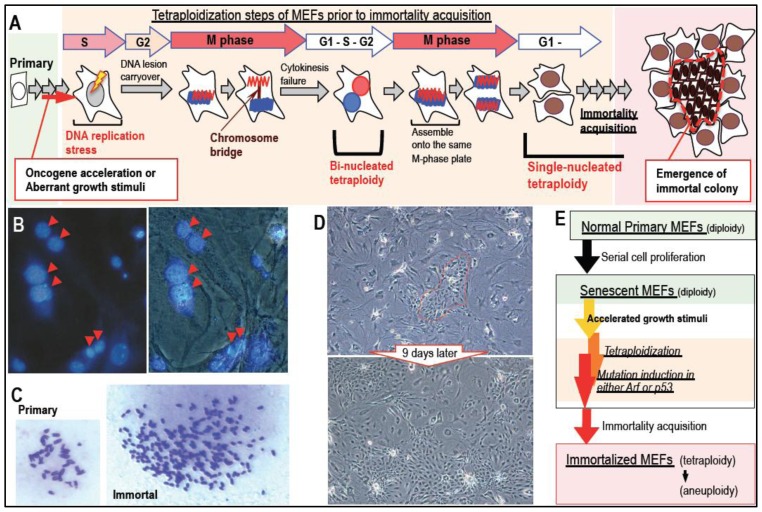
Process of tetraploidization and immortality acquisition. (**A**) Model of the tetraploidization process with the subsequent emergence of immortality. Oncogenic stress induced by oncogene activation or aberrant growth stimulation leads to the accumulation of DNA replication stress-associated lesions in cells and accelerated entry into S phase. These DNA lesions are not efficiently repaired, and are thereby carried over into M phase and mediate chromosomal-bridge formation, which leads to tetraploidy development after the failure of cytokinesis. The resulting tetraploidy is initially associated with two nuclei (bi-nucleated tetraploidy) until the next M phase, in which chromosomes assemble on the same M phase plate to develop single-nucleated tetraploidy in the subsequent G1 phase; (**B**) MEFs in the senescent stage show accumulated bi-nucleated tetraploidy. Nuclei are stained with DAPI. Red double arrowheads indicate bi-nucleated tetraploid cells; (**C**) Images showing the induction of chromosomal instability after immortality acquisition; (**D**) The emergence of immortality is usually observed in morphologically senescent MEFs. Colonies of immortalized MEFs are generated from morphologically senescent MEFs (flattened and enlarged morphology). Such immortalized MEFs eventually become predominant; (**E**) Steps leading to senescence and immortalization of normal MEFs are schematically described along with the chromosomal status at each step.

In response to oncogenic DNA replication stress-associated lesions, cells activate damage checkpoint responses and downstream barrier reactions, such as senescence and apoptosis induction [55,56,64]. However, induced DNA lesions are not efficiently repaired and, thus, are often carried over into M phase without completion of the repair process (Figure 2A). This causes chromosome-bridge formation upon missegregation of chromosomes during mitosis, which leads to cytokinesis failure and tetraploidy development [33]. Although the induced tetraploidy is initially bi-nucleated, this is only transient because the chromosomes of two nuclei assemble on the same M phase plate and then segregate to each side, leading to the formation of two single-nucleus tetraploids at the following G1 phase [33] (Figure 2A). In fact, prior to the acquisition of immortality, senescent MEFs cultured using the 3T3 protocol [46] often show chromosome-bridge formation and accumulation of bi-nucleated tetraploid cells [33,54] (Figure 2B). Immortalized MEFs are subsequently generated, which have a mutated Arf/p53 module (either in Arf or p53) [32] in association with tetraploidy [33] (Figures 1A and 2C). In these immortalized MEFs, the loss of senescent morphology and the acquisition of primary-like phenotypes in terms of morphology and growth activity (Figure 2D) become predominant (Figure 2D). During these processes, tetraploidization occurs in rarely-growing senescent cells. In contrast, the emergence of immortality is associated with the loss of senescent characteristics; the resulting immortalized cells, therefore, gain growth activity and an altered morphology.

## 3. Mutation Induction during the Development of Tetraploidy

In addition to developing genomic instability, cancer cells accumulate a number of mutations [24], although only a few may be required for carcinogenesis. The mutations essential for carcinogenesis include at least the following two types: (a) tissue-specific mutations, such as mutations in the APC regulation module in the colon [65]; and (b) mutations in the Arf/p53 module, which are likely to be common mutations in malignant cancer cells [32]. Importantly, unlike the tissue specific mutations observed in cancer, the Arf/p53 module is also mutated in cells immortalized/transformed *in vitro* [32]. This indicates that the Arf/p53 module is involved in cellular regulatory pathways that are common to various tissues.

Although the mechanisms underlying the induction of mutations in the Arf/p53 module are still unclear, an *in vitro* model suggests that they are the direct consequence of tetraploidization [47] (Figure 2E). In fact, whereas immortality acquisition in normal MEFs occurs only with tetraploidy and mutations in the Arf/p53 module, *p53*-knockout MEFs immortalize while diploid [47]. This suggests that tetraploidization is necessary for immortalization of wild-type MEFs with mutations in the Arf/p53 module, but not for the development of immortality (as long as p53 function is lost). In addition, wild-type MEFs cannot develop immortality with genome stability [47] under conditions in which the Arf/p53 module is continuously functional. These lines of evidence indicate that tetraploidy development directly contributes to mutation induction in the Arf/p53 module, and tetraploidy develops prior to the acquisition of immortality in barely-proliferating normal MEFs.

## 4. Arf/p53 Module-Dependent Quiescent Cellular Status

**Figure 3:**
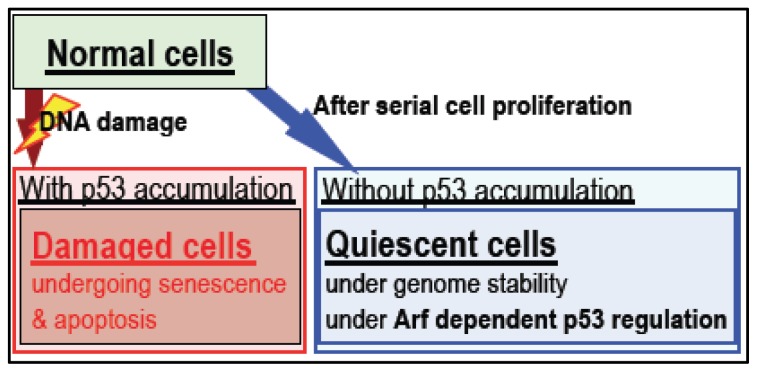
*p53* plays two distinct roles in the acute DNA damage response and the establishment of quiescent cellular status. Whereas damaged cells show acute responses and often undergo premature senescence and apoptosis, normal cells spontaneously become growth-arrested under the regulation of Arf/p53 under conditions in which a quiescent state is induced. This quiescent cellular status is under the functional regulation of the Arf/p53 module but, unlike in cells undergoing acute damage responses, does not involve p53 accumulation.

Similar to the process of development of malignant cancers, mutations in the Arf/p53 module are widely induced during immortality acquisition *in vitro* [32,33,47]. However, the exact role of Arf/p53 in the suppression of cellular transformation is still unclear. A recent study shows that most of the direct transcription targets of p53 are associated with the acute DNA damage response, but are not required for tumor suppression (Figure 3), suggesting two separate functions for p53 [66]. In addition, *Arf* and *p53*, the two most frequently mutated genes in cancer, are part of the same MDM2-mediated regulatory module and are mutated in a mutually exclusive manner [4]. This strongly suggests that the essential role of p53 in cancer suppression is dependent on *Arf* regulation, and that cells acquire immortality only in the presence of mutations in *Arf* and *p53*. By contrast, p53-dependent acute damage responses are even observed in cancer cells, indicating that cancer cells without *p53* mutations are more sensitive to DNA damaging agents than *p53*-mutated cancer cells. Thus, unlike *Arf*-independent *p53* activation (e.g., through checkpoint responses), the role of *p53* in cancer suppression is likely to be regulated by *Arf*.

Because Arf and p53 are critical tumor suppressors, *Arf*- and *p53*-knockout (KO) mice show a significantly increased predisposition to cancer [67,68]. By contrast, transgenic mice with additional single gene copies of *Arf* and *p53* are characterized by cancer suppression and an extended lifespan [32], indicating normal regulation of the *Arf/p53* genes. However, unlike mice that show functional *Arf* and *p53* regulation, transgenic mice with hyper-active p53 with no MDM2-binding site show a reduced lifespan and premature aging [69–71]. In these mice, *p53* is not regulated by Arf because the normal MDM2-mediated inhibition of Arf is absent. Thus, unlike its stress response-associated function, under normal conditions the Arf/p53 module functions simultaneously to extend lifespan and suppress cancer. Importantly, the two functions of p53 are distinguished by differences in p53 levels; *i.e.*, (1) hardly-detectable levels of p53 under normal conditions are associated with extended lifespan and cancer suppression; and (2) accumulated p53 is associated with premature aging and with senescent and/or apoptotic cells [55,56], which are also characterized by *p53* overexpression [72].

*Arf* is coded on the same gene locus as another cancer suppressor, *INK4a*. Therefore, mutations in *Arf* may affect *INK4a* expression. However, unlike I*NK4a*-KO mice, *Arf*-KO mice show spontaneous tumor development, as do *p53*-KO mice [73]. Furthermore, unlike *INK4a*-KO MEFs, which senesce in a similar manner to wild-type MEFs, primary Arf-KO MEFs directly acquire immortality in a manner similar to *p53*-KO MEFs [73]. Thus, unlike *INK4a*, *Arf* (along with *p53*) is involved in essential cellular regulatory functions that induce growth-arrest.

## 5. Cellular Quiescence Is Produced with Arf/p53-Dependent H2AX Diminution

The exact contribution of the Arf/p53 module to growth arrest is unclear. We recently determined that normal cells show decreased H2AX levels after serial proliferation under the regulation of the Arf/p53 module, which contributes to growth arrest [47]. In agreement with this, cells in which *H2AX* was either knocked-down or knocked-out show severe growth retardation [74–81]. Importantly, decreased H2AX levels are also observed in adult mouse organs, such as liver, spleen, and pancreas, in which cells rarely proliferate [47]. On the other hand, the mechanisms underlying *H2AX* downregulation are absent in cancer cells due to mutations in the Arf/p53 module.

**Figure 4:**
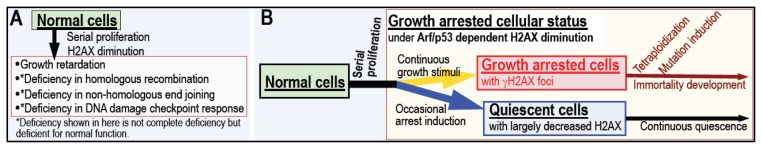
Arf/p53-dependent decrease of H2AX contributes to the characteristics of the growth-arrested state. Normal cells in a growth-arrested state due to a decrease in Arf/p53-dependent H2AX levels show similar characteristics to cells in which *H2AX* is either knocked-out or knocked-down (**A**). However, these growth-arrested states are discriminated by either a marked decrease in H2AX levels or by the accumulation of γH2AX (**B**), resulting in either continuous quiescence or tetraploidization and immortalization, respectively.

Intriguingly, certain characteristics of senescent cells and growth-arrested cells may be the result of Arf/p53-dependent H2AX downregulation, because cells without H2AX show the same characteristics, including growth retardation and defects in DNA damage repair and checkpoint responses (Figure 4A) [74–81]. Therefore, cells showing Arf/p53-dependent H2AX downregulation are sensitive to accelerated growth stimulation by exogenous stresses, which leads to the development of tetraploidy (Figure 2A). This includes the effects of preserving the quiescent state and the risk of developing genomic instability [47] (Figure 4B). During cell maintenance (with occasional growth-arrest), cells enter a quiescent state, in which *H2AX* is largely downregulated. This quiescent cellular state is preserved under the functional regulation of the Arf/p53 module and the maintenance of genome stability [47]. On the other hand, cells under continuous growth stimulation accumulate DNA replication stress-associated lesions and exhibit γH2AX because they undergo accelerated entry into S phase [47]. In addition, cells showing a reduced level of H2AX are defective in DNA damage repair and DNA damage checkpoint responses [47]. These unrepairable DNA lesions are not efficiently removed and are carried over into M phase, causing tetraploidization and, subsequently, inducing mutations in the Arf/p53 module (Figure 2A), which lead to recovery of *H2AX* expression and growth activity and the acquisition of immortality. Taken together, growth arrest in normal cells can be separated into two states [47]: (1) a continuously quiescent state with largely downregulated *H2AX* under genome stability maintenance; and (2) a state at risk of developing tetraploidy with γH2AX accumulation. Although the regulatory mechanisms that lead to the different cellular states are still unclear, our recent results demonstrate that growth stimulation is involved [47].

**Figure 5:**
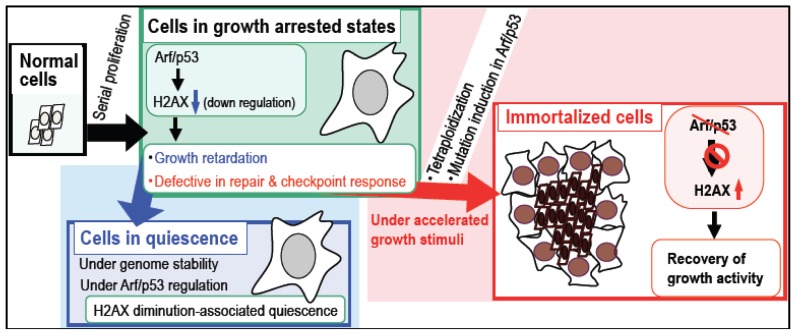
Life cycle of normal cells. Normal cells show high expression of H2AX in the early stages along with active proliferation, followed by growth retardation. As senescence progresses (after serial cell proliferation), H2AX levels decrease under the regulation of the Arf/p53 module. Because such a growth-arrested state is a consequence of Arf/p53-dependent H2AX downregulation, growth-arrested cells are also defective in DNA damage repair and checkpoint responses. These cells, which are also at risk of genomic instability under conditions of exogenous growth stimulation, subsequently develop tetraploidy and mutations in the Arf/p53 module. This leads to a recovery of H2AX levels and growth activity, resulting in the acquisition of immortality. By contrast, cells that maintain genomic stability preserve their quiescent status. Because normal cells undergo growth-arrest associated with downregulation of H2AX, which is regulated by the Arf/p53 module, exogenous growth stimulation is critical for either homeostasis or for the development of tetraploidy and immortality.

After serial cell proliferation, cells enter a growth-arrested state associated with Arf/p53-dependent downregulation of *H2AX* (Figure 5). By contrast, cells subjected to stress by oncogenes and growth stimuli are characterized by persistent exhibition of γH2AX [55,56], which is also a characteristic of senescent cells and aging organs where it is induced by a variety of stresses [14–22]. However, Arf/p53-dependent downregulation of *H2AX* is often abrogated during the development of cancer, as well as during *in vitro* cellular transformation associated with mutations in the Arf/p53 module [74–81].

To suppress cellular transformation, normal cells generally enter a growth-arrested state and downregulate *H2AX* in an Arf/p53-dependent manner; immortality is, therefore, inevitably associated with Arf/p53 mutations, which are triggered by genomic instability. The mechanism(s) underlying the involvement of the Arf/p53 module in *H2AX* downregulation is still unclear. *H2AX* is probably not the direct target of p53 because the promoter region of *H2AX* does not contain a p53-binding site. In addition, Arf/p53 might not be the only mechanism by which H2AX is downregulated because miR24, which reduces *H2AX* in terminally-differentiated blood cells [82], is unlikely to be the direct target of p53 [83]. These are some of the issues that need to be addressed in future studies.

## 6. Conclusions

After serial cell proliferation, normal cells eventually undergo growth arrest (Figure 5). The cells may then become quiescent; a state in which cells show largely diminished levels of H2AX under the regulation of the Arf/p53 module. However, this state is abrogated by exogenous growth stimuli, which cause accelerated entry into S phase and DNA replication stress. Because cells with reduced levels of H2AX are defective in DNA damage repair and checkpoint responses, the unrepairable DNA lesions are often carried over into M phase and induce tetraploidy. Although most of these tetraploid cells are still growth arrested and show a senescent morphology, immortalized cells will appear with the Arf/p53 module-mutation, which develops in association with tetraploidization. Thus, normal cells generally undergo quiescence when H2AX is downregulated by the Arf/p53 module under conditions of genome stability, in which cells maintain quiescence. However, in the presence of exogenous growth stimulation, the development of genomic instability (tetraploidy) and mutations in the Arf/p53 module lead to the loss of the quiescence and ultimately result in cellular transformation.

These recent findings illustrate the critical role of H2AX downregulation in the establishment of a growth-arrested cellular state. The phenotypes of the cells in this state are often expressed in association with Arf/p53-dependent downregulation of H2AX; these include deficiencies in DNA repair and checkpoint responses, and an increased risk of genomic instability. Therefore, to avoid cellular transformation, genome stability must be maintained in cells after they reach the growth-arrested state characterized by H2AX downregulation.
